# New Approaches in Motor Intervention for Infants Aged 0–2 Years with or at High Risk of Unilateral or Bilateral Cerebral Palsy: A Systematic Review

**DOI:** 10.3390/children13060762

**Published:** 2026-05-30

**Authors:** Laura Beccani, Monica Valle, Sara Damilano, Francesco Venturelli, Massimo Vicentini, Olivia Vecchi, Silvia Faccioli

**Affiliations:** 1 Pediatric Rehabilitation Unit, Azienda Unità Sanitaria Locale IRCCS di Reggio Emilia, Viale Risorgimento 80, 42123 Reggio Emilia, Italy; 2 Rehabilitation Service, Public Health and Paediatric Sciences Department, A.O.U. Città della Salute e della Scienza, Regina Margherita Children Hospital, 10126 Turin, Italy; 3 Centro Puzzle, Via Cimabue 2, 10137 Turin, Italy; 4 Epidemiology Unit, Azienda Unità Sanitaria Locale IRCCS di Reggio Emilia, Via Amendola 2, 42122 Reggio Emilia, Italy

**Keywords:** cerebral palsy, early motor intervention, infants

## Abstract

**Highlights:**

**What are the main findings?**

- Early motor interventions for infants with or at high risk of cerebral palsy share common therapeutic principles despite heterogeneity in intervention models.
- There is a trend toward clinically meaningful improvements in motor and developmental outcomes after all types of intervention, although methodological limitations remain.

**What are the implications of the main findings?**

- Early, family-centered, and task-specific interventions should be prioritized during the first year of life to maximize neuroplastic potential.
- Standardization of outcome measures and inclusion of younger infants are essential to advance evidence-based early intervention.

**Abstract:**

**Background/Objectives:** Early motor intervention is increasingly recognized as a critical component in the management of infants with, or at high risk of, cerebral palsy (CP). This systematic review aimed to synthesize recent evidence on early motor interventions in infants aged 0–2 years and to identify current gaps in knowledge. **Methods:** We performed a systematic literature review across PubMed, Embase, CINAHL, and Scopus. Studies published between 1 January 2020 and 5 October 2024 were included. Eligible studies were Randomized Controlled Trials (RCTs), non-randomized trials, and cohort studies that involved infants aged 0–2 years diagnosed with CP or classified as at high risk of CP who received early motor interventions targeting motor outcomes. Study selection and data extraction were executed by two independent reviewers following standardized protocols. The Rob2 Checklist was used to assess the risk of bias. This systematic review protocol was registered on PROSPERO with the ID 506784. **Results:** Six articles representing four RCTs were included. Although intervention protocols varied, shared therapeutic principles emerged across studies. Most participants were approximately 12 months old; only one study included infants younger than 3 months, highlighting limited evidence in the earliest detectable risk period. No consistent superiority of experimental interventions over standard care was observed; however, notable within-group improvements in motor and developmental domains were reported across both study arms. Major limitations included heterogeneity of outcome measures, limited use of CP-specific standardized tools, and insufficient assessment of functional and goal-based outcomes. **Conclusions**: Current evidence indicates a shift toward meaningful, family-centered early motor interventions, emphasizing active participation and parental involvement. Five core principles were identified: early initiation, task specificity, active experience, guided support, and engagement of both the child and caregivers. Future research should focus on earlier intervention timing, standardized outcome measures, and caregiver-related outcomes to optimize early intervention strategies during critical developmental windows.

## 1. Introduction

Globally, cerebral palsy (CP) represents the most frequent physical disability emerging during early childhood, affecting approximately 2 out of every 1000 live births [1]. This term delineates a group of permanent neurodevelopmental conditions that affect motor control and posture, leading to restrictions in activity. The underlying etiology involves non-progressive disturbances that take place within the embryonic or early infant brain. The resulting clinical picture is ‘persistent but not immutable’; as the child grows, the interaction between the static brain injury, the maturation of the central nervous system, and environmental factors leads to a continuous evolution of posture and movement patterns [2].

Due to its diverse etiology, phenotypic presentation, and wide spectrum of severity, CP is recognized as a highly heterogeneous and clinically complex condition. These characteristics necessitate a multidisciplinary and comprehensive approach to improving the health profile as defined by the International Classification of Functioning, Disability and Health (ICF) [3] and accordingly by international guidelines and care pathways [4].

In 2017, Novak et al. [5] published international guidelines for the early detection of CP, establishing that it can be accurately predicted before the corrected age of six months. Diagnosis requires a standardized neuromotor assessment, including the Prechtl Qualitative Assessment of General Movements (GMs) and the Hammersmith Infant Neurological Examination (HINE), along with neonatal magnetic resonance imaging (MRI) and a clinical history of risk factors. When a clinical diagnosis is suspected but not confirmed, it is crucial to define the infant as being at “high risk for cerebral palsy” until a definitive diagnosis is made. This classification requires motor dysfunction (an essential criterion) plus at least one of two additional criteria: abnormal neuroimaging or a clinical history of risk factors.

The clinical value of establishing an early diagnosis rests on the immediate initiation of timely interventions aimed at optimizing infant brain plasticity. Neuroplasticity is a process involving adaptive structural and functional changes in the brain, enabling neural networks to modify their activity through growth and reorganization in response to intrinsic or extrinsic stimuli.

Infants possess a significant, yet fragile, potential for neuroplasticity. Although early theories like the ‘Kennard Principle’ posited a linear advantage for younger patients, revised models recognize that functional outcomes depend on lesion characteristics and environmental factors [6,7]. This reinforces the existence of a critical developmental window, making timely therapeutic intervention essential to leverage early brain potential.

Neuroplasticity, however, cannot be defined a priori as either positive or negative. It requires early and appropriate intervention to promote adaptive characteristics. Neuromotor interventions encouraging movement serve as potential tools to guide neuronal circuits in a beneficial direction. Such interventions should employ experience-dependent plasticity, motivation, and attention to create rewarding experiences that lead to spontaneous and consistent practice [8]. In 2021, Morgan et al. [9] defined the key principles of effective early intervention: early initiation, specificity, parental support, and caregiver participation. The intervention must also ensure a balanced activity to sustain brain reorganization akin to physiological development—this is the challenging task for both therapists and families. In contrast, generic, passive motor interventions are often less effective and may even be counterproductive, potentially driving plasticity in a maladaptive direction [10].

Despite the necessity of personalizing interventions for individual children, the trajectory of gross motor development usually plateaus near 90% of its maximum potential by age five, highlighting the 0–2 age range as the window of most significant improvement [11]. The Italian recommendations distinguish “caretaking”—a primary, pivotal, multidisciplinary intervention spanning the patient’s lifespan—from “cure-taking,” which involves the delivery of specific therapeutic interventions [3]. During early childhood, interventions should be focused on fostering fundamental developmental acquisitions. In infants at high risk for or diagnosed with CP, motor interventions are commonly initiated [9], aiming to promote major adaptive functions such as posture, walking, manipulation, and visual system development [12].

Despite the growing interest in early intervention, there is still a lack of detailed descriptions of habilitative and rehabilitative approaches available to children diagnosed with or at risk for CP at an early age. Aside from interventions targeting manipulative function in hemiplegic patients, there is a strong need to examine the existing literature to address the latest clinical knowledge needs and identify potential gaps. While the principle of early intervention is well established, scientific evidence supporting specific approaches remains limited. Notably, even the most recent international guidelines acknowledge that the quality of evidence across many intervention domains remains variable, with several recommendations being downgraded to ‘conditional’ due to a reliance on interpolated data from non-CP populations [9]. This suggests a persistent operational gap regarding the precise technical protocols, dosages, and specific clinical potentials of emerging motor treatments. Ongoing neuroscience research highlights the crucial link between timely, diagnosis-appropriate intervention and a child’s functional and motor prognosis in the short, medium, and long term. This underscores the need for further analysis of various habilitative and rehabilitative approaches and the most recent studies in the field to provide clinicians with more granular and evidence-based operational tools.

Accordingly, the aim of this systematic review is to examine recent evidence on early motor interventions for infants aged 0–2 years with or at high risk of cerebral palsy, with the objectives of identifying common therapeutic principles, evaluating reported motor outcomes, and highlighting current gaps in the literature to inform future research and clinical practice.

## 2. Materials and Methods

The Preferred Reporting Items for Systematic Reviews and Meta-Analyses (PRISMA) 2020 guidelines were utilized as the foundational framework to conduct this systematic review [13]. The review question was: “What are the motor interventions in infants aged 0–2 years with cerebral palsy or at high risk of cerebral palsy?”

The PICOs method was used to summarize the review question:

P (Patient and Problem)—Infants 0–2 years with cerebral palsy or high risk of cerebral palsy.

I (Intervention)—Early motor intervention, defined as motor intervention started before the second year of life.

C (Comparison)—Standard/usual care.

O (Outcome)—Improvement in motor skills, such as hand function, gait and sitting position, measured with valid and standardized assessment tools (e.g., GMFM-88, AHA).

S (Study design)—Randomized Controlled Trials (RCTs), non-randomized trials, and cohort studies with comparators.

### 2.1. Eligibility Criteria

Inclusion criteria were: articles with a population of infants aged 0–24 months, diagnosed with CP or “at high risk of”; studies of mixed age groups were included if participants’ age was lower than 6 years and if the data of participants aged from 0 to 2 years were reported separately; papers with infants who received early motor intervention; studies with standardized assessment tools evaluating motor skills; articles published between 1 January 2020 and 5 October 2024.

To complement and update the comprehensive analysis on early cerebral palsy interventions (0–2 years) published by Morgan et al. (2021) [9], we restricted our search to the subsequent timeframe. This approach allowed us to identify and discuss the specific literature gaps that have arisen since their review.

Exclusion criteria were articles where the intervention was medical, pharmaceutical, or surgical and papers not written in the English or Italian languages.

### 2.2. Information Source and Search Strategy

PubMed, Embase, CINAHL, and Scopus were used to conduct the search. With the help of a medical librarian and according to the review question, search strategies were specifically built for each source (Supplementary File S1—Search strategies).

We restricted our search strategy to English-language studies published between 2020 and 2024 to map out the most recent insights. The final electronic search was performed on 5 October 2024.

### 2.3. Selection Process

Rayyan [14] was used to detect and resolve duplicates and for the screening of titles and abstracts. The screening and selection of eligible studies were carried out independently by two authors (M.V., S.D.). In the event of disagreements, resolution was achieved via mutual consensus or through consultation with a third independent investigator (L.B.).

### 2.4. Data Collection Process and Synthesis Methods

A data extraction spreadsheet was used to summarize the main characteristics of the included studies. For each included study, data collection covered the following fields: title, authors, publication year, study design, participant details, intervention characteristics, comparison groups, primary and secondary outcomes (including related assessment tools), main results, and additional notes. Data extraction was performed independently by a pair of reviewers (M.V., S.D.). Discrepancies were resolved by consensus or by involving a third reviewer (L.B.) to verify and finalize the extraction matrix.

### 2.5. Data Items

#### 2.5.1. Types of Studies

Randomized Controlled Trials (RCTs), non-randomized trials, and cohort studies with comparators were included in this review.

#### 2.5.2. Participant Characteristics

Eligibility criteria targeted infants aged 0 to 2 years with a confirmed diagnosis or a high risk of cerebral palsy who underwent early motor intervention protocols. A child is defined as ‘at high risk’ of CP if any of the following criteria are present: absent fidgety movements in the General Movements Assessment, positive brain imaging, diagnosis of hypoxic–ischemic encephalopathy.

#### 2.5.3. Outcomes

The targeted outcomes focused primarily on functional and motor development alongside other domains of pediatric evolution, whereas parameters evaluating parents or caregivers fell outside the scope of this review. All the measures that demonstrate changes in motor function are included. We did not limit inclusion to specific motor outcome measurements.

#### 2.5.4. Interventions and Comparators

Early motor intervention regarding improving motor skills provided by physical, occupational or neurodevelopmental disorder therapists was the focus of the review. Some examples of such interventions are constraint-induced movement therapy, bimanual therapy, occupational therapy, and movement therapy with the aim of improving motor function in infants with cerebral palsy.

Articles with non-specific interventions on motor performance, such as infant massage or aquaticity, were excluded.

All types of comparator interventions were considered. Studies that have at least one control group which is exposed to standard/usual care or any other alternative intervention strategies were included.

### 2.6. Study Risk of Bias Assessment

Methodological bias for the Randomized Controlled Trials (RCTs) was quantified and screened in accordance with the RoB 2.0 assessment criteria. Although the use of the ROBINS-I tool had been pre-specified in the study protocol for the evaluation of non-randomized intervention studies, it was not ultimately applied as no such studies met the inclusion criteria.

### 2.7. Effect Measures

While the calculation of measures of change—specifically the between-group standardized mean difference (SD) for each outcome (post-intervention minus baseline) comparing the intervention group versus controls—was originally planned, this analysis was ultimately not feasible due to the high degree of heterogeneity found across the included literature.

### 2.8. Registration

In accordance with methodological transparency, this systematic review was officially documented in the PROSPERO registry in 2024: https://www.crd.york.ac.uk/prospero/display_record.php?ID=CRD42024506784 (accessed on 6 February 2024).

## 3. Results

**Figure 1:**
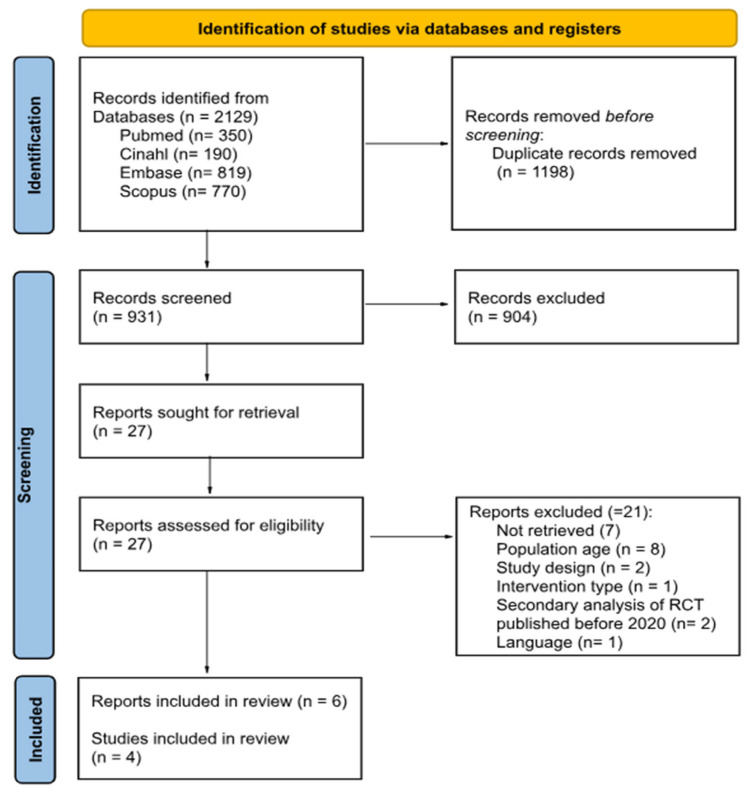
PRISMA 2020 flow diagram.

In Figure 1 the PRISMA flow chart is represented. Details regarding the completed PRISMA checklist can be found in Supplementary File S2. Table 1 provides a comprehensive summary of the extracted data architecture. In addition to essential bibliographic information (authors and titles), the table details the clinical characteristics of the cohorts, such as participant numbers and age distribution. The specific components of the early motor interventions and their corresponding comparison arms are fully described. Regarding clinical efficacy, the table maps out the targeted outcomes, their measurement scales, and the major findings, which have been separated into primary trial results and secondary analyses where relevant. Lastly, the methodological quality is contextualized by listing the internal strengths and limitations reported in the original studies. Reports excluded are summarized in Supplementary File S4. The search of the four databases yielded a total of 2129 reports. After the screening, six papers were finally included. Nonetheless, two of them present further data or a secondary analysis of previously included RCTs.

A brief description of the studies is provided below.

Benfer et al. [15] published a study conducted on 153 infants aged 12 to 40 weeks corrected age (CA) at risk for CP. The Learning through Everyday Activities with Parents (LEAP-CP) program was an early intervention delivered by peers at home and included targeted goal-directed training with cognitive learning games and support for caregivers. An equal dose of health advice (HA) was the comparison. Mobility function was the main infant outcome of the study, and it was evaluated with the mobility domain of the Pediatric Evaluation of Disability Inventory–Computer Adaptive Test (PEDI-CAT mobility). Caregiver mental health was the other primary outcome. Secondary outcomes investigated infant development. In ambulant children with CP, the LEAP-CP protocol demonstrated greater efficacy in enhancing motor skills, corroborating established evidence on targeted, goal-directed training. Conversely, regarding parental psychological outcomes, the investigators revealed no significant differences between the LEAP-CP intervention and dose-matched HA when evaluated via the Depression Anxiety and Stress Scale.

Cemali et al. [16] presented sensory integration training plus conventional physiotherapy in infants aged 12–18 months with CP and CVI (Cortical Vision Impairment). The control group received only conventional physiotherapy. Sensory modalities and motor function were evaluated through the Test of Sensory Function in Infants (TSFI) and the Alberta Infant Motor Scale (AIMS), respectively. Both cohorts exhibited highly significant longitudinal improvements from pre- to post-test assessments in total TSFI and AIMS scores (*p* < 0.001). Furthermore, while the intervention group displayed a higher upward trajectory in mean TSFI scores relative to the control group, this numerical advantage did not achieve statistical significance. No differences were observed in post-intervention AIMS scores between groups. While the addition of sensory integration to standard physiotherapy showed favorable trends in sensory processing for infants with CVI and CP, definitive superiority over standard care alone was not established.

Both studies by Hielkema et al. 2020 [17,18] referred to the same RCT: one reported neuromotor, cognitive, and behavioral outcomes [17], while the other focused on infants’ functional and family outcomes [18]. Participants were infants aged 0–9 months at risk for bilateral and unilateral CP. The intervention was COPCA (coping with and caring for infants with special needs) and the comparison was with TIP (Traditional Infant Physiotherapy). The COPCA program was created in the early 2000s in the Netherlands and is family-centered. The theoretical foundation rests on the neuronal group selection theory, which fosters motor variability by engaging the infant in an active learning framework driven by trial-and-error exploration. Therapists educate parents to be autonomous in coping with everyday life situations and making decisions about child development [21]. The main outcome of the first paper [17] was Infant Motor Profile (IMP) and the outcome measures were IMP itself, the Albert Infant Motor Scale (AIMS), the Gross Motor Function Measure (GMFM) and the Bayley Scale of Infants Development—Psychomotor Developmental Index, second edition (Bayley II). The study indicated that both COPCA and typical infant physiotherapy generate comparable neurodevelopmental trajectories over a one-year period in infants at high risk for CP. Furthermore, the discussion highlights the potential necessity of merging active elements from different rehabilitative paradigms to enhance early intervention outcomes.

In the second paper, the main outcomes were activities and participation of infants, family empowerment and quality of life. The outcome measurements were PEDI (Pediatric Evaluation of Disability Index), FES (Family Empowerment Scale), ITQOL (Infant and toddler quality of life questionnaire) and CBS Quality of Life. The study indicated that COPCA and TIP exert comparable effects on both child and family outcomes. Interestingly, while the data demonstrated beneficial correlations between specific COPCA components and family-related endpoints, no significant associations were found between the intervention elements and child-specific developmental outcomes.

Harbourne et al. [19] and Babik et al. [20] referred to the same RCT. Participants in the study were infants aged 7–16 months with different types of motor delay (CP, at risk of CP, unknown origin). The population was stratified into two groups by movement ability, designed to achieve equivalent groups. The intervention was Sitting Together and Reaching to Play (START-Play) plus UC-EI (Usual Care Early Intervention) and comparison was with UC-EI. The START-Play intervention was created by drawing from previous research and the grounded cognition theory of child development [22,23]. Its goal was to enhance motor skills, specifically sitting and reaching, and cognitive abilities in children with neuromotor delays. The intervention focused on developing four key cognitive concepts—body and object affordances, object permanence, means–end relationships, and joint attention—through engaging motor activities and social interactions [24]. In the first paper, outcome measurements were GMFM, reach video assessment, Assessment of Problem Solving in Play (APSP), and Bayley III. The investigators concluded that the START-Play protocol could accelerate the acquisition of reaching, problem-solving, and cognitive and fine-motor skills in the short term for young infants presenting with severe motor delays compared to UC-EI. Conversely, supplementary START-Play added to UC-EI did not appear to yield superior motor or cognitive benefits over standard care for infants with milder motor impairments. Additionally, the study suggested that incorporating concepts of embodied cognition into early intervention strategies—as operationalized in START-Play—can effectively catalyze cognitive and motor development in infants with pronounced motor delays relative to traditional early intervention frameworks.

The second paper focused on reaching-related exploratory behaviors. To evaluate reaching behaviors, video recordings were analyzed by blinded assessors who codified six distinct motor profiles: total, unimanual, bimanual, ventral, and openhanded contact, alongside visual fixation (looking). Based on these observations, the authors concluded that rehabilitation programs prioritizing the early cultivation of reaching skills through problem-solving frameworks—such as the START-Play intervention—represent a valuable strategy for children presenting with major motor delays.

### Risk of Bias

**Figure 2:**
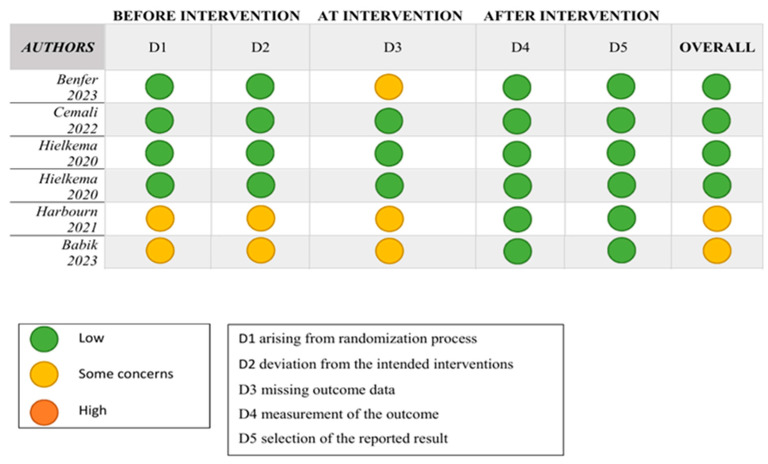
Risk of bias assessed with the Rob2 Checklist [15,16,17,18,19,20].

An overview of the methodological quality and risk of bias across the selected trials is illustrated in Figure 2.

The Rob2 Checklist [25] was used to assess the risk of bias. The RCTs “Learn 2 Move” [17,18] and Cemali et al. [16] presented a low risk of bias across all checklist domains. All four RCTs considered in this review employed appropriate randomization procedures (column D1). Regarding deviation from the intended intervention (column D2), concerns about bias were noted for the RCT “Start Play” [19,20] as it was a single-blind study. The evaluator was blinded, but it was not specified whether the families were blinded or not. Concerning missing outcome data (column D3), the rating of ‘some concerns’ (moderate risk) for attrition in the “Start Play” and Benfer studies was based on the statistical consequences of dropout as explicitly acknowledged by the authors themselves. The “Start Play” intervention experienced a 20.5% dropout rate. As such, the analysis of the effect might be partially invalidated, as the groups analyzed at the end of the study had fewer participants than initially required. For this reason, column D3 was rated as presenting a moderate risk of bias. Benfer [15], in estimating the sample size of n = 142, accounted for a 10% dropout rate. A total of 153 participants were enrolled at the start of the study, with 32 lost to follow-up. The dropout rate was therefore slightly higher than originally anticipated but remained below 20%. A moderate risk of bias was also assigned to this study. These author-reported consequences were used as objective criteria for identifying potential bias.

## 4. Discussion

We identified six manuscripts, reporting the results of four high-quality Randomized Controlled Trials (RCTs). They highlighted the clinical relevance of early intervention in infants at risk of CP. These studies, calibrated for a specific rehabilitative population, exhibit great homogeneity in terms of participant age, primarily targeting infants under 12 months, exactly the population of interest for infant early intervention. However, a notable limitation is that only one study [15] included infants as young as 12 weeks old, leaving the “newborn detectable risk” population underrepresented [5].

In all studies except one [15], standard care was mediated by an experienced pediatric therapist, with interventions designed to enhance motor skills within the child’s home environment. This indicates a shift toward the “just-right challenge” concept, where therapy is skill-directed within meaningful, familiar contexts, abandoning outdated notions of passivity and generic interventions.

The experimental interventions incorporated standard care with added parental support, underscoring the growing recognition of parental involvement as a cornerstone of early rehabilitative intervention. The literature increasingly examines parent engagement and empowerment, which will likely shape future training for pediatric rehabilitation professionals, including within university curricula.

While none of the studies showed a significant difference between the experimental and standard care groups regarding primary motor function outcomes, significant improvements were observed across all cohorts. However, these results should be interpreted with caution; the lack of a statistically significant difference may not necessarily confirm the high efficacy of standard care but rather reflect the inherent challenges of these trials. Factors such as the high degree of clinical heterogeneity in the population and the relatively small sample sizes often confound between-group comparisons, making it difficult to isolate specific treatment effects or establish a clear superiority of one approach over another. Our findings regarding the lack of clear ‘add-on’ value are highly consistent with recent high-level evidence syntheses in the field [9,26].

A key limitation identified is the use of outcome rating scales that lack normative values for children with CP, such as the PEDI and Bayley scales, which primarily measure gross milestones rather than specific motor functions. Additionally, no studies included analytical rating scales for manipulation (HAI [27]) or posture and standing (Segmental Assessment of Trunk Control Satco [28]), nor goal-based scales (GAS [29]).

Children with CP reach 90% of their motor potential by age five, with the most development occurring between 0 and 2 years [11]. Early intervention supports development, participation, and autonomy, aligning with ICF principles [26]. While early intervention has been a cornerstone of clinical practice for decades, more research is needed to define the most effective treatments. Recent guidelines and the “risk of CP” category have significantly advanced early detection and intervention, but further studies are required to refine best practices and maximize rehabilitation outcomes.

### 4.1. Key Concepts in Early Motor Intervention

**Figure 3:**
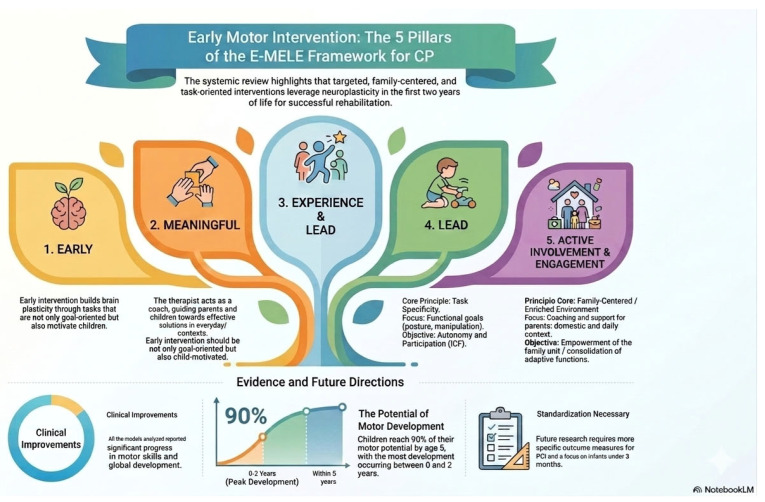
Graphical abstract for E-MELE. Image generated using AI (Notebook LM).

Building upon the evidence identified in this review and integrating established neurodevelopmental principles, we propose the E-MELE framework (Figure 3). This conceptual model serves as an interpretive tool to help healthcare professionals translate clinical evidence into practice by focusing on five key pillars:

- Early (time and age matter): It is widely recognized that the first two years of life represent a critical period for the development of adaptive functions [15,30]. To achieve these functions, it is crucial for therapists to sustain the attachment process between mother and infant. This bond helps the child establish homeostatic balance with their environment, a necessary step before initiating a proper skills-directed treatment [17,31].
- Meaningful (goal- or task-specific, motor learning principle, the just-right challenge): The therapist should design activities that capture the child’s attention, interest, and emotional involvement, combining hands-off and hands-on strategies of intervention while providing a sense of satisfaction [17,20]. In doing so, they will promote the acquisition of foundational motor functions such as posture, manipulation, gait, and vision [16,20].
- Experience (use it or lose it, use it and improve it, enriched environment): The therapist should enable the child to actively experience adaptive functions, so that they can be stably consolidated across all life contexts [18]. These contexts will be adapted to the child’s abilities, thus generating repeated and intensive experiences [15,19,32,33].
- Lead (coaching, affordances): The therapist plays a key role in guiding the child and their parents to identify the most appropriate solutions within a given task and to achieve effective and efficient outcomes [16,17,21]. This is accomplished by carefully managing the environment and the objects involved, with particular attention to the affordances they provide [34,35].
- Engagement (child active, self-initiated activity and empowerment): The therapist facilitates the child’s participation and engagement to sustain child motivation and attention. The therapist, aided by peer supporters, should also transfer all the skills described above to the parents [15,18,36].

### 4.2. Future Direction (Pilot Studies and Protocols)

A review of excluded studies revealed four relevant RCT protocols describing interventions such as GAME (Goals–Activity–Motor Enrichment), e-HABIT-ILE, GO-PLAY, LEAP-CP, and infant-mCIMT/BIT. Most involve home-based settings and parent coaching. Six pilot studies analyzed tele-rehabilitation approaches or video coaching, with two studies exploring rehabilitation tools like sensor-equipped toys. Many studies also investigated caregiver well-being outcomes. These works primarily focus on tele-rehabilitation, sensor-based tools, and parent-coaching models, reinforcing the field’s movement toward intensive, home-based, and family-centered care.

### 4.3. Limitations of the Articles

Although the evaluated trials demonstrated robust quality with a ‘low’ risk of bias, or merely ‘some concerns’, several methodological limitations still warrant careful consideration. Small sample sizes, heterogeneous outcome measures, and limited use of CP-specific, functional, and goal-based assessments restrict comparability across studies. Finally, caregiver-related outcomes, participation measures, and long-term follow-up were rarely included, limiting insight into broader functional and psychosocial impacts.

### 4.4. Limitations of the Review

A potential language bias must be acknowledged, as the search strategy was confined to articles published in English. Additionally, because our protocol targeted a restricted developmental window, relevant evidence from clinical trials featuring broader pediatric age groups may have been overlooked. Furthermore, due to the high clinical and methodological heterogeneity of the included interventions, a formal meta-analysis was not performed, as a qualitative narrative synthesis was deemed more appropriate to provide a meaningful overview of the current evidence.

## 5. Conclusions

This systematic review highlights a clear evolution in early motor intervention for infants with or at high risk of cerebral palsy toward meaningful, activity-based, and family-centered approaches. Despite heterogeneity in intervention models, five core principles were consistently identified: early initiation, task specificity, active experience, guided support, and engagement of both the infant and caregivers. While current evidence does not demonstrate the superiority of any specific intervention protocol, a trend toward improved motor and developmental outcomes was observed across the included studies. However, given the methodological heterogeneity and the lack of a clear advantage over standard care, these findings should be interpreted with caution, and the definitive efficacy of early motor interventions remains to be established through more rigorous research. Bridging the gap between early detection and intervention remains a key challenge. Future research should focus on earlier implementation, standardized outcome measures, and the inclusion of functional and caregiver-related outcomes to optimize intervention strategies during critical periods of neurodevelopmental plasticity.

## Figures and Tables

**Table 1:** Main characteristics of the included studies.

Study	Participants	Intervention	Comparison	Outcomes	Measurement of Outcomes	Main Results or Findings	Limits and Strengths Declared by the Authors
Efficacy of Early Intervention for Infants with Cerebral Palsy in an LMIC: An RCT Benfer et al. [15] 2023	Infants 12–40 weeks corrected age at risk for CP 153 randomized (77–76) 33 dropouts Effect size 142	LEAP-CP (Learning through Everyday Activities with Parents- CP) 15 home visits by a peer trainer	HA (health advice) Equal dose by peer trainer	1. Mobility function/caregiver mental health 2. Infant development	1. PEDI-CAT mobility, DASS 2. COPM, PDMS-2, BSID-III, Near Detection Scale, nutritional status, HINE, HOME, PSOC	Primary and secondary outcomes improve in both arms without a difference between groups. When stratified for GMFCS (96 infants), children with GMFCS I and II in Leap-cp scored better than HA on PEDI-CAT mobility and HINE. Intervention groups improve parent reported motor skills (Pedi-cat) and neurological status (Hine) in ambulatory children with cp. Ambulatory children have better response reported than non-ambulatory.	An absence of true active control arm. Greater severity of the sample in the Leap group. LMIC populations skewed toward poorer motor function and comorbidities. Use of GMFCS at 18 months is less accurate than use in older children. The study did not exclude children with significant comorbidities. Real-world setting in low- and middle-income countries. Both study arms had clinically and statistically important improvements for all outcomes. Peer supporters instill hope and facilitate service involvement.
The Effectiveness of Sensory Integration Interventions on Motor and Sensory Functions in Infants with Cortical Vision Impairment and Cerebral Palsy: A Single Blind Randomized Controlled Trial. Cemali et al. [16] 2022	Infants 12–18 months with CP and CVI 36 randomized (17–17) 2 dropouts Effect size 34	Sensory integration training + conventional physiotherapy 4 sessions of 45 min/week for 8 weeks in outpatient setting (dedicated room) by a physiotherapy	Conventional physiotherapy 2 sessions of 45 min/week for 8 weeks in outpatient setting	1. Sensory and motor functions	1. TSFI, AIMS	Both groups improved significantly. Intervention group in TSFI (except for subheading oculomotor control) scored better than controls. AIMS improved in the same way between groups.	Infants participating were not homogeneous for type of cp and gender. Duration of intervention was limited. Use of protocol and checklist in the intervention group to systematize the intervention. The study will guide clinicians in understanding the importance of sensory processing in infants with CVI.
LEARN2MOVE 0–2 years, a randomized early intervention trial for infants at very high risk of cerebral palsy: neuromotor, cognitive, and behavioral outcome. Hielkema et al. [17] 2020 LEARN2MOVE 0–2 years, a randomized early intervention trial for infants at very high risk of cerebral palsy: family outcome and infant’s functional outcome. Hielkema et al. [18] 2020	Infants 0–9 months at risk for CP (bilateral and unilateral) 43 randomized (23–20) 4 dropouts Effect size 38	COPCA (Coping with and caring for infants with special needs) Once a week, 30–60 min per session, at home by pediatric physiotherapist	TIP (traditional infant physiotherapy) Equal dose	Neuromotor, cognitive and behavioral outcomes 1. Activities and participation of infants 2. Family empowerment outcomes 3. Quality of life	1. IMP 2. AIMS, GMFM-66/GMFM-88, BSID II, CBCL, video recording session analyzed with Groningen Observer Protocol 2.0 1. FES, NOSIK, UCL 2. PEDI, VABS 3. ITQOL, CBS-list	*Primary trial results:* Infants’ neuromotor, cognitive and behavioral outcomes improve significantly over time. The improvement was similar in both groups. *Secondary analysis:* 1. The effect of COPCA and TIP on the family outcomes was similar. 2. In both intervention groups PEDI and VABS scores increased significantly over time 3. Similar effect in both groups but in COPCA group some domains scores changed significantly over time. Caregivers were more satisfied with general health. They felt less emotionally worried and less restricted.	Clinicians need good measurement tools because some scales have been developed for the general population and only few are specifically for CP. A long recruitment period which increases risk of contamination between groups. The intervention dosage may have affected outcomes. A longitudinal evaluation of early infants at very high risk of cp. A detailed process analysis of the intervention contents. Brain imaging is available which could be able to investigate relations between lesions and outcome over time. A small sample size. Not all measurements were appropriate for young age (es. Nosi-k). Questionnaires were lower than infants’ motor measures (selection bias). Evaluation of both infants and family outcomes. A large battery of measurement. A longitudinal study (12 months).
START-Play Physical Therapy Intervention Impacts Motor and Cognitive Outcomes in Infants with Neuromotor Disorders: A Multisite Randomized Clinical Trial. Harbourne et al. [19] 2021 The Effect of START-Play Intervention on Reaching-Related Exploratory Behaviors in Children with Neuromotor Delays: A Secondary Analysis of a Randomized Controlled Trial. Babik et al. [20] 2023	Infants 7–16 months with motor delay (CP, risk for CP, unknown origin) Stratification in 2 groups (Severity and Mild) 112 randomized (57–55) 23 dropouts Effect size 152	START-Play (sitting together and reaching to play) + UC-EI (usual care—early intervention) Twice weekly 40–60 min home visit for 12 weeks + UC	UC-EI (usual care-early intervention) 4, 5 sessions per week for 12 weeks (Severity 5,5 Mild 2,5 sessions for week)	1. Sitting, reaching 2. roblem solving, global developmental outcome Reaching-related exploratory behaviors	1. GMFM, reach video assessment 2. APSP, Bayley III Videos of the reaching assessment (10 behavioral outcomes)	*Primary trial results:* Aggregating across severity level there were no differences between groups. Stratified for severity, they found different results over time. SHORT PERIOD 1. There were no significant effects. 2. There were significant positive effects for START-P for severity infants in APSP and Bayley cognitive and motor domain. 3. Significant positive effects in UC for mild infants in APSP and Bayley communication domain. LONG PERIODS 1. Significant positive effects for START-P for Severity infants in reaching frequency. 2. Significant positive effects for START-P for Severity infants in the Bayley fine-motor domain. 1.2. No significant effects in mild infants. *Secondary analysis:* BASELINE In UC group, severity infants were higher than START-P. SHORT PERIOD Mild Infants in START-P catch up with their peers. LONG PERIOD Mild Infants in START-P improved in reaching and looking objects.	There was underpower caused by a dropout. The decision to stratify groups was not planned when determining the power of the study. Testing problem assessment in infants is difficult. Dose of intervention was not equal between groups. Contained short and long follow-up. Stratification could help to better understand the impact of intervention across different samples. Both interventions incorporate literature suggestions such as a natural environment, coaching, focusing on whole-child ability, and meaningful routines. Current analyses could lead to spurious inferences. Significant baseline differences among groups. Interventions focused on reaching skills with the contexts of problem solving should be considered for children with significant motor delay.

Legend: CP (Cerebral Palsy), PEDI-CAT (Pediatric Evaluation of Disability Inventory–Computer Adaptive Test), DASS (Depression Anxiety and Stress Scale), COPM (Canadian Occupational Performance Measure) PDMS-2 (Peabody Developmental Motor Scales—second edition) BSID-II, III (Bayley Scales of Infant Development—second or third edition), HINE (Hammersmith International Neurological Examination), HOME (Home Observation for Measurement of the Environment) PSOC (Parenting Sense of Competence Scale), GMFCS (Gross Motor Function Classification System), CVI (Cortical vision impairment), TSFI (Test of Sensory Function in Infants), AIMS (Alberta Infant Motor Scale), IMP (Infant Motor Profile), GMFM-66/GMFM-88 (Gross Motor Function Measure), (Child Behavior Checklist), FES (Family Empowerment Scale), NOSI-K (Nijmeegse Ouderlijke Stress Index), UCL (Utrechtse Coping List), PEDI (Pediatric Evaluation of Disability Index), VABS (Vineland Adaptive Behavior Scale), ITQOL (Infant and toddler quality of life questionnaire), CBS-list (Caregiver Quality of Life Questionnaire), APSP (Assessment of Problem Solving in Play). Underline is used to distinguish strengths from limitations.

## Data Availability

The data presented in this study are available upon request from the corresponding author due to privacy reasons.

## Supplementary Materials

The following supporting information can be downloaded at: https://www.mdpi.com/article/10.3390/children13060762/s1, File S1: Search strategies. File S2: PRISMA checklist. File S3: PRISMA abstract checklist. File S4: Reports excluded.

## Abbreviations

The following abbreviations are used in this manuscript:

CP	Cerebral Palsy
RCT	Randomized Controlled Trial
PRISMA	Preferred Reporting Items for Systematic reviews and Meta-Analyses
ICF	International Classification of Functioning, Disability and Health
GMs	General Movements
