# Supplementary material for: New Approaches in Motor Intervention for Infants Aged 0–2 Years with or at High Risk of Unilateral or Bilateral Cerebral Palsy: A Systematic Review
Source: Children (Basel). 2026 May 30;13(6):762. doi: 10.3390/children13060762 (PMC13296985; doi:10.3390/children13060762)
Supplement: Supplementary file 1 [file children-13-00762-s001.zip › File S1 search strategies.pdf]

Pubmed

("Cerebral Palsy"[Mesh] OR cerebral-pals*[tiab] OR little-disease*[tiab] OR little's-disease*[tiab] OR spastic-diplegi*[tiab] OR CP[tiab] OR "Hemiplegia"[Mesh] OR hemiplegi*[tiab] OR monoplegi*[tiab] OR hemiparalysis[tiab] OR spastic[tiab] OR hypotonic[tiab] OR atonic[tiab] OR dyskinetic[tiab] OR athetoid[tiab] OR monoplegi*[tiab] OR congenital[tiab] OR rolandic[tiab] OR quadriplegic-infantile[tiab] OR mixed[tiab] OR dystonic-rigid[tiab] OR "Hypoxia-Ischemia, Brain"[Mesh] OR HIE[tiab] OR hypoxicischemic-encephalopath*[tiab] OR ischemic-hypoxic-encephalopath*[tiab] OR neonatal-encephalopath*[tiab] OR neonatal-strok*[tiab] OR intraventricular-hemorrhag*[tiab] OR intraventricular-haemorrhag*[tiab] OR IVH[tiab] OR periventricular-leucomalaci*[tiab] OR periventricular-leukomalaci*[tiab] OR PVL[tiab] OR arterial-ischemic-strok*[tiab] OR arterial-ischaemic-strok*[tiab] OR middle-cerebral-artery-embolus*[tiab] OR brain-hypoxia-ischemi*[tiab] OR brain-ischemia-hypoxi*[tiab] OR hypoxic-ischemic-encephalopath*[tiab] OR anoxic-ischemic-encephalopath*[tiab] OR cerebral-hypoxia-ischemi*[tiab] OR cerebral-ischemia-hypoxi*[tiab] OR brain-anoxia-ischemi*[tiab] OR brain-ischemia-anoxi*[tiab] OR cerebral-anoxia-ischemi*[tiab] OR cerebral-ischemia-anoxi*[tiab] OR "Cerebral Infarction"[Mesh] OR mca-infarct*[tiab] OR cerebral-infarct*[tiab] OR brain-infarct*[tiab] OR cerebral-artery-thrombosis*[tiab] OR hydrocephal*[tiab] OR cerebral-strok*[tiab]) AND ("Infant"[Mesh] OR neonat*[tiab] OR newborn[tiab] OR infant*[tiab] OR baby[tiab] OR babies[tiab] OR toddler*[tiab] OR premat*[tiab] OR "young child*"[tiab]) AND ("Occupational Therapy"[Mesh] OR "Physical Therapy Specialty"[Mesh] OR "Physical Therapists"[Mesh] OR "Physical Therapy Modalities"[Mesh] OR "Restraint, Physical"[Mesh] OR "Exercise Therapy"[Mesh] OR "Early Intervention, Educational"[Mesh] OR occupation-therap*[tiab] OR occupational-therap*[tiab] OR physiotherap*[tiab] OR physical-therap*[tiab] OR ergotherap*[tiab] OR constraint-induced-movement-therap*[tiab] OR constraint-induced-therap*[tiab] OR physical-restraint*[tiab] OR physical-immobiliz*[tiab] OR neuro-developmental-therap*[tiab] OR motor-training[tiab] OR NDT[tiab] OR neurodevelopmental-therap*[tiab] OR bobath[tiab] OR neurophysiotherap*[tiab] OR exercise*[tiab] OR early-intervent*[tiab] OR head-start-program*[tiab]) AND ("Upper Extremity"[Mesh] OR "Lower Extremity"[Mesh] OR "Motor Skills"[Mesh] OR "Motor Skills Disorders"[Mesh] OR "Gait"[Mesh] OR "Gait Disorders, Neurologic"[Mesh] OR motor-function*[tiab] OR motor-skill*[tiab] OR motor-develop*[tiab] OR developmental-coordinat*[tiab] OR skill-acquis*[tiab] OR gross-motor*[tiab] OR fine-motor[tiab] OR upper-limb-function*[tiab] OR lower-limb-function*[tiab] OR hand-function*[tiab] OR foot-function*[tiab] OR movement*[tiab] OR gait[tiab] OR "Treatment Outcome"[Mesh] OR motor-outcome*[tiab] OR treatment-outcome*[tiab] OR physical-outcome*[tiab] OR neurodevelopmental-outcome*[tiab] OR neuro-developmental-outcome*[tiab] OR treatment-effective*[tiab] OR rehabilitation-outcome*[tiab] OR treatment-efficac*[tiab]) AND ("2020/01/01"[Date - Entry] : "2024/10/04"[Date - Entry])

350 results

Embase

('cerebral palsy'/exp OR 'cerebral pals*':ti,ab,kw OR 'little disease*':ti,ab,kw OR 'spastic diplegi*':ti,ab,kw OR cp:ti,ab,kw OR 'hemiplegia'/exp OR hemiplegi*:ti,ab,kw OR monoplegi*:ti,ab,kw OR hemiparalysis:ti,ab,kw OR spastic:ti,ab,kw OR hypotonic:ti,ab,kw OR atonic:ti,ab,kw OR dyskinetic:ti,ab,kw OR athetoid:ti,ab,kw OR congenital:ti,ab,kw OR rolandic:ti,ab,kw OR 'quadriplegic infantile':ti,ab,kw OR mixed:ti,ab,kw OR 'dystonic rigid':ti,ab,kw OR 'hypoxic ischemic encephalopathy'/exp OR hie:ti,ab,kw OR 'hypoxicischemic encephalopath*':ti,ab,kw OR 'ischemic hypoxic encephalopath*':ti,ab,kw OR 'neonatal encephalopath*':ti,ab,kw OR 'neonatal strok*':ti,ab,kw OR 'intraventricular hemorrhag*':ti,ab,kw OR

'intraventricular haemorrhag*':ti,ab,kw OR ivh:ti,ab,kw OR 'periventricular leucomalaci*':ti,ab,kw OR 'periventricular leukomalaci*':ti,ab,kw OR pvl:ti,ab,kw OR 'arterial ischemic strok*':ti,ab,kw OR 'arterial ischaemic strok*':ti,ab,kw OR 'middle cerebral artery embolus*':ti,ab,kw OR 'brain hypoxia ischemi*':ti,ab,kw OR 'brain ischemia hypoxi*':ti,ab,kw OR 'hypoxic ischemic encephalopath*':ti,ab,kw OR 'anoxic ischemic encephalopath*':ti,ab,kw OR 'cerebral hypoxia ischemi*':ti,ab,kw OR 'cerebral ischemia hypoxi*':ti,ab,kw OR 'brain anoxia ischemi*':ti,ab,kw OR 'brain ischemia anoxi*':ti,ab,kw OR 'cerebral anoxia ischemi*':ti,ab,kw OR 'cerebral ischemia anoxi*':ti,ab,kw OR 'brain infarction'/exp OR 'mca infarct*':ti,ab,kw OR 'cerebral infarct*':ti,ab,kw OR 'brain infarct*':ti,ab,kw OR 'cerebral artery thrombosis*':ti,ab,kw OR hydrocephal*:ti,ab,kw OR 'cerebral strok*':ti,ab,kw) AND ('infant'/exp OR neonat*:ti,ab,kw OR newborn:ti,ab,kw OR infant*:ti,ab,kw OR baby:ti,ab,kw OR babies:ti,ab,kw OR toddler*:ti,ab,kw OR premat*:ti,ab,kw OR 'young child*':ti,ab,kw) AND ('occupational therapy'/exp OR 'physiotherapy'/exp OR 'physiotherapist'/exp OR 'physical restraint'/exp OR 'kinesiotherapy'/exp OR 'early childhood intervention'/exp OR 'occupation therap*':ti,ab,kw OR 'occupational therap*':ti,ab,kw OR physiotherap*:ti,ab,kw OR 'physical therap*':ti,ab,kw OR ergotherap*:ti,ab,kw OR 'constraint induced movement therap*':ti,ab,kw OR 'constraint induced therap*':ti,ab,kw OR 'physical restraint*':ti,ab,kw OR 'physical immobiliz*':ti,ab,kw OR 'neuro developmental therap*':ti,ab,kw OR 'motor training':ti,ab,kw OR ndt:ti,ab,kw OR 'neurodevelopmental therap*':ti,ab,kw OR bobath:ti,ab,kw OR neurophysiotherap*:ti,ab,kw OR exercise*:ti,ab,kw OR 'early intervent*':ti,ab,kw OR 'head start program*':ti,ab,kw) AND ('upper limb'/exp OR 'lower limb'/exp OR 'motor performance'/exp OR 'psychomotor disorder'/exp OR 'gait'/exp OR 'neurologic gait disorder'/exp OR 'motor function*':ti,ab,kw OR 'motor skill*':ti,ab,kw OR 'motor develop*':ti,ab,kw OR 'developmental coordinat*':ti,ab,kw OR 'skill acquis*':ti,ab,kw OR 'gross motor*':ti,ab,kw OR 'fine motor':ti,ab,kw OR 'upper limb function*':ti,ab,kw OR 'lower limb function*':ti,ab,kw OR 'hand function*':ti,ab,kw OR 'foot function*':ti,ab,kw OR movement*:ti,ab,kw OR gait:ti,ab,kw OR 'treatment outcome'/exp OR 'motor outcome*':ti,ab,kw OR 'treatment outcome*':ti,ab,kw OR 'physical outcome*':ti,ab,kw OR 'neurodevelopmental outcome*':ti,ab,kw OR 'neuro developmental outcome*':ti,ab,kw OR 'treatment effective*':ti,ab,kw OR 'rehabilitation outcome*':ti,ab,kw OR 'treatment efficac*':ti,ab,kw) AND [01-01-2020]/sd NOT [04-10-2024]/sd

819 results

Cinahl

((MH "Cerebral Palsy") OR TI (cerebral-pals* OR little-disease* OR little's-disease* OR spastic-diplegi* OR CP) OR AB (cerebral-pals* OR little-disease* OR little's-disease* OR spastic-diplegi* OR CP)

OR

(MH "Hemiplegia") OR TI (hemiplegi* OR monoplegi* OR hemiparalysis OR spastic OR hypotonic OR atonic OR dyskinetic OR athetoid OR congenital OR rolandic OR quadriplegic-infantile OR mixed OR dystonic-rigid) OR AB (hemiplegi* OR monoplegi* OR hemiparalysis OR spastic OR hypotonic OR atonic OR dyskinetic OR athetoid OR congenital OR rolandic OR quadriplegic-infantile OR mixed OR dystonic-rigid)

OR

(MH "Hypoxia-Ischemia, Brain") OR TI (HIE OR hypoxicischemic-encephalopath* OR ischemic-hypoxic-encephalopath* OR neonatal-encephalopath* OR neonatal-strok* OR intraventricular-hemorrhag* OR intraventricular-haemorrhag* OR IVH OR periventricular-leucomalaci* OR periventricular-leukomalaci* OR PVL OR arterial-ischemic-strok* OR arterial-ischaemic-strok* OR middle-cerebral-artery-embolus* OR

brain-hypoxia-ischemi* OR brain-ischemia-hypoxi* OR hypoxic-ischemic-encephalopath* OR anoxic-ischemic-encephalopath* OR cerebral-hypoxia-ischemi* OR cerebral-ischemia-hypoxi* OR brain-anoxia-ischemi* OR brain-ischemia-anoxi* OR cerebral-anoxia-ischemi* OR cerebral-ischemia-anoxi*) OR AB (HIE OR hypoxicischemic-encephalopath* OR ischemic-hypoxic-encephalopath* OR neonatal-encephalopath* OR neonatal-strok* OR intraventricular-hemorrhag* OR intraventricular-haemorrhag* OR IVH OR periventricular-leucomalaci* OR periventricular-leukomalaci* OR PVL OR arterial-ischemic-strok* OR arterial-ischaemic-strok* OR middle-cerebral-artery-embolus* OR brain-hypoxia-ischemi* OR brain-ischemia-hypoxi* OR hypoxic-ischemic-encephalopath* OR anoxic-ischemic-encephalopath* OR cerebral-hypoxia-ischemi* OR cerebral-ischemia-hypoxi* OR brain-anoxia-ischemi* OR brain-ischemia-anoxi* OR cerebral-anoxia-ischemi* OR cerebral-ischemia-anoxi*)

OR

(MH "Cerebral Infarction") OR TI (mca-infarct* OR cerebral-infarct* OR brain-infarct* OR cerebral-artery-thrombosis* OR hydrocephal* OR cerebral-strok*) OR AB (mca-infarct* OR cerebral-infarct* OR brain-infarct* OR cerebral-artery-thrombosis* OR hydrocephal* OR cerebral-strok*))

AND

((MH "Infant") OR TI (neonat* OR newborn OR infant* OR baby OR babies OR toddler* OR premat* OR “young child*”) OR AB (neonat* OR newborn OR infant* OR baby OR babies OR toddler* OR premat* OR “young child*”))

AND

((MH "Occupational Therapy") OR (MH "Physical Therapy") OR (MH "Physical Therapists") OR (MH "Restraint, Physical") OR (MH "Therapeutic Exercise") OR (MH "Early Intervention+") OR TI (occupation-therap* OR occupational-therap* OR physiotherap* OR physical-therap* OR ergotherap* OR constraint-induced-movement-therap* OR constraint-induced-therap* OR physical-restraint* OR physical-immobiliz* OR neuro-developmental-therap* OR motor-training OR NDT OR neurodevelopmental-therap* OR bobath OR neurophysiotherap* OR exercise* OR early-intervent* OR head-start-program*) OR AB (occupation-therap* OR occupational-therap* OR physiotherap* OR physical-therap* OR ergotherap* OR constraint-induced-movement-therap* OR constraint-induced-therap* OR physical-restraint* OR physical-immobiliz* OR neuro-developmental-therap* OR motor-training OR NDT OR neurodevelopmental-therap* OR bobath OR neurophysiotherap* OR exercise* OR early-intervent* OR head-start-program*))

AND

((MH "Upper Extremity") OR (MH "Lower Extremity") OR (MH "Motor Skills") OR (MH "Motor Skills Disorders") OR (MH "Gait") OR (MH "Gait Disorders, Neurologic") OR TI (motor-function* OR motor-skill* OR motor-develop* OR developmental-coordinat* OR skill-acquis* OR gross-motor* OR fine-motor OR upper-limb-function* OR lower-limb-function* OR hand-function* OR foot-function* OR movement* OR gait) OR AB (motor-function* OR motor-skill* OR motor-develop* OR developmental-coordinat* OR skill-acquis* OR gross-motor* OR fine-motor OR upper-limb-function* OR lower-limb-function* OR hand-function* OR foot-function* OR movement* OR gait)

OR

(MH "Treatment Outcomes") OR TI (motor-outcome* OR treatment-outcome* OR physical-outcome* OR neurodevelopmental-outcome* OR neuro-developmental-outcome* OR treatment-effective* OR rehabilitation-outcome* OR treatment-efficac*) OR AB (motor-outcome* OR treatment-outcome* OR physical-outcome* OR neurodevelopmental-outcome* OR neuro-developmental-outcome* OR treatment-effective* OR rehabilitation-outcome* OR treatment-efficac*))

AND

(EM 20200101-20241004)

190 results

Scopus

TITLE-ABS-KEY (cerebral-pals* OR little-disease* OR little's-disease* OR spastic-diplegi* OR CP OR hemiplegi* OR monoplegi* OR hemiparalysis OR spastic OR hypotonic OR atonic OR dyskinetic OR athetoid OR congenital OR rolandic OR quadriplegic-infantile OR mixed OR dystonic-rigid OR HIE OR hypoxicischemic-encephalopath* OR ischemic-hypoxic-encephalopath* OR neonatal-encephalopath* OR neonatal-strok* OR intraventricular-hemorrhag* OR intraventricular-haemorrhag* OR IVH OR periventricular-leucomalaci* OR periventricular-leukomalaci* OR PVL OR arterial-ischemic-strok* OR arterial-ischaemic-strok* OR middle-cerebral-artery-embolus* OR brain-hypoxia-ischemi* OR brain-ischemia-hypoxi* OR hypoxic-ischemic-encephalopath* OR anoxic-ischemic-encephalopath* OR cerebral-hypoxia-ischemi* OR cerebral-ischemia-hypoxi* OR brain-anoxia-ischemi* OR brain-ischemia-anoxi* OR cerebral-anoxia-ischemi* OR cerebral-ischemia-anoxi* OR mca-infarct* OR cerebral-infarct* OR brain-infarct* OR cerebral-artery-thrombosis* OR hydrocephal* OR cerebral-strok*) AND TITLE-ABS-KEY (neonat* OR newborn OR infant* OR baby OR babies OR toddler* OR premat* OR “young child*”) AND TITLE-ABS-KEY (occupation-therap* OR occupational-therap* OR physiotherap* OR physical-therap* OR ergotherap* OR constraint-induced-movement-therap* OR constraint-induced-therap* OR physical-restraint* OR physical-immobiliz* OR neuro-developmental-therap* OR motor-training OR NDT OR neurodevelopmental-therap* OR bobath OR neurophysiotherap* OR exercise* OR early-intervent* OR head-start-program*) AND TITLE-ABS-KEY (motor-function* OR motor-skill* OR motor-develop* OR developmental-coordinat* OR skill-acquis* OR gross-motor* OR fine-motor OR upper-limb-function* OR lower-limb-function* OR hand-function* OR foot-function* OR movement* OR gait OR motor-outcome* OR treatment-outcome* OR physical-outcome* OR neurodevelopmental-outcome* OR neuro-developmental-outcome* OR treatment-effective* OR rehabilitation-outcome* OR treatment-efficac*) AND PUBYEAR > 2019 AND PUBYEAR < 2025

770 results
