# Supplementary material for: New Approaches in Motor Intervention for Infants Aged 0–2 Years with or at High Risk of Unilateral or Bilateral Cerebral Palsy: A Systematic Review
Source: Children (Basel). 2026 May 30;13(6):762. doi: 10.3390/children13060762 (PMC13296985; doi:10.3390/children13060762)
Supplement: Supplementary file 1 [file children-13-00762-s001.zip › File S4 summarized excluded.pdf]

Author & Year	Study title	Reason for Exclusion
**Araneda L et al 2024**	Changes Induced by Early Hand-Arm Bimanual Intensive Therapy Including Lower Extremities in Young Children With Unilateral Cerebral Palsy: A Randomized Clinical Trial.	Population age
**Boyd R et al 2022**	REACH: Randomised comparison trial of rehabilitation early for congenital hemiplegia	Report non retrieved
**Boyd R et al 2023**	Efficacy of infant friendly Baby-CIMT and Baby-BIM in a randomised trial of home-based parent delivered early intervention for infants at risk of unilateral cerebral palsy	Report non retrieved
**Grinde K et al 2020**	Repeated episodes of pediatric constraint induced movement therapy with a gross motor training component: A prospective cohort study.	Population age
**Hwang YS et al 2020**	Effects of Modified Constraint-Induced Movement Therapy in Real-World Arm Use in Young Children with Unilateral Cerebral Palsy: A Single-Blind Randomized Trial	Population age
**Kaittan NM et al 2020**	Low power glove for hand functioning analysis in children with cerebral palsy	Population age
**Klevberg GL et al 2020**	Development of Hand Use with and Without Intensive Training Among Children with Unilateral Cerebral Palsy in Scandinavia.	Population age
**Lowing K et al 2020**	Do infants at risk of developing cerebral palsy or other neurodevelopmental disorders learn what they practice?	Secondary analysis of RCT published before 2020
**Mattern-Baxter K et al 2020**	Low-Intensity vs High-Intensity Home-Based Treadmill Training and Walking Attainment in Young Children With Spastic Diplegic Cerebral Palsy	Population age
**Palee S et al 2020**	Goal-directed therapy improves clinical outcomes and quality of life in children with cerebral palsy: A randomized controlled trial	Population age
**Palomo R et al 2023**	“Shall we start? Ready, set, go!” Towards early intervention in infants with unilateral cerebral palsy: Preliminary results	Report non retrieved
**Prosser LA et al 2022**	iMOVE (Intensive Mobility with Variability and Error) Trial for Toddlers with Cerebral Palsy	Population age
**Prosser LA et al 2023**	Motor training for young children with cerebral palsy: A single-blind randomized controlled trial.	Report non retrieved

**Sanches E et al 2024**	Early intensive rehabilitation reverses locomotor disruption, decrease brain inflammation and induces neuroplasticity following experimental Cerebral Palsy	Intervention type
**Santina Z et al 2021**	Acceptability, feasibility and fidelity of a neonatal rehabilitation program for infants born preterm at high risk for cerebral palsy	Report non retrieved
**Sakzewski L et al 2023**	Development of the impaired hand during the first 15 months of life in infants with unilateral cerebral palsy	Report non retrieved
**Scott K et al 2023**	Therapist fidelity in a multi-site randomized comparative efficacy trial of Baby-CIMT and Baby-BIM for infants with unilateral cerebral palsy	Report non retrieved
**Tebani A et al 2021**	Early Intervention in Cerebral Palsy and Beyond.	Study design
**Verhaegh A PM et al 2023**	Multisensory Stimulation and Priming (MuSSAP) in 4-10 Months Old Infants with a Unilateral Brain Lesion: A Pilot Randomised Clinical Trial.	Study design
**Yuan A et al 2023**	Effects of goals-activity-motor enrichment therapy on gross and fine motor function in infants at high risk of cerebral palsy	Language
**Zipp GP et al 2021**	Commentary on "Improvements in Muscle Strength Are Associated With Improvements in Walking Capacity for Young Children With Cerebral Palsy: A Secondary Analysis".	Secondary analysis of RCT published before 2020
